# Regression of multifocoal in transit melanoma metastases after palliative resection of dominant masses and 2 years after treatment with ipilimumab

**DOI:** 10.1186/s40425-017-0259-9

**Published:** 2017-07-18

**Authors:** Raphael B. Moreira, Lana Hamieh, Evisa Gjini, Ana Lako, Katherine M. Krajewski, Charles H. Yoon, Patrick A. Ott

**Affiliations:** 1 Department of Medical Oncology, Dana Farber Cancer Institute, Brigham and Women’s Hospital, and Harvard Medical School, Boston, MA USA; 2 Center for Immuno-Oncology, Dana-Farber Cancer Institute, Boston, MA USA; 3 Department of Imaging, Dana Farber Cancer Institute, Brigham and Women’s Hospital Harvard Medical School, Boston, MA USA; 4 Department of Surgery, Brigham and Women’s Hospital, Harvard Medical School, Boston, MA USA; 5 Melanoma Disease Center & Center for Immuno-Oncology, Dana Farber Cancer Institute Harvard Medical School, Boston, MA 02215 USA

**Keywords:** PD-1, Immune checkpoint blockade, Antibody therapy, Melanoma, Immunotherapy

## Abstract

**Background:**

Spontaneous regression of metastatic melanoma and delayed responses more than one year after treatment with ipilimumab are rarely seen.

**Case presentation:**

Here, we present the case of a patient with in transit metastases from cutaneous melanoma on his right lower extremity who achieved complete regression of all metastatic lesions 13 months after the first of two consecutive palliative resections of dominant masses and more than two years after treatment with ipilimumab.

**Conclusion:**

The exact cause of our patient’s sudden onset of tumor regression remains speculative. We hypothesize that the operative trauma followed by the postoperative infections augmented an innate immune response.

## Background

Immune checkpoint blockade using monoclonal antibodies have been approved by the US Food and Drug Administration (FDA) to treat patients with advanced melanoma. We describe a case involving a patient that received ipilimumab, a monoclonal antibody against cytotoxic T-lymphocyte associated protein 4 (CTLA-4). CTLA-4 is a cell surface receptor that negatively regulates the immune response and its blockade can influence anti-tumor T cell activity. Ipilimumab showed a survival benefit in Phase III trials involving patients with advanced melanoma [1, 2]. Durable tumor responses in patients with advanced melanoma treated with ipilimumab yielded a plateau in the survival curve at 21% starting at 3 years from study initiation [3]. Here, we present a patient with multifocal in-transit melanoma metastases who achieved spontaneous regression two years after completion of ipilimumab.

## Case presentation

**Fig. 1:**
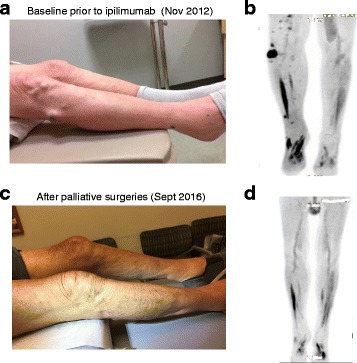
In transit metastases as evident clinically and on PET. **a**, **b** At baseline (October/November 2012). **c**, **d** Two years after the 2nd of 2 palliative surgical resections (September 2016)

In October of 2012 an 84-year-old man with a history of coronary artery disease, COPD, hypertension, and venous insufficiency presented with multiple cutaneous nodules on his right leg. The lesions had been growing in size over the preceding 3 years (Fig. 1a). An excisional biopsy was performed and revealed a malignant melanoma with focal necrosis. The lesion was described as purple, tender, 2.7 × 2.5 × 1.5 cm in size. A PET/CT of the entire body demonstrated a dominant soft tissue mass lateral to the right fibular head with numerous additional soft tissue nodules extending from the right mid thigh anteriorly to the level of the ankle, compatible with multiple cutaneous and subcutaneous melanoma metastases (Fig. 1b). There was no evidence for distant metastatic disease. BRAF^V600^ status was found to be wild-type. The patient was not deemed a candidate for hyperthermic isolated limb perfusion due to peripheral vascular disease and the perceived very high risk for development of distant metastatic disease. Between December 2012 and February 2013 the patient received 4 cycles of the anti-CTLA-4 monoclonal antibody ipilimumab, at the standard dose of 3 mg/kg given once every 3 weeks, which he intially tolerated well except for intermittent low-grade diarrhea and fatigue. In April 2013, he developed anemia with a hemoglobin of 6.7 g/dl requiring transfusions. An extensive work up including bone marrow biopsy suggested pure red cell aplasia, which is rare however has been previously described after treatment with CTLA-4 blockade [4], as the most likely etiology. The patient was treated with a pulse of dexamethasone for 4 days at 1 mg/kg-day, with no change in his transfusion requirements and no rise of the reticulocyte count, then intravenous immunoglobulin (IVIg), with no reticulocytosis and no normalization of his hemoglobin. His cytogenetics showed 5/20 cells positive for del(5q), consistent with myelodysplastic syndrome and he therefore received a course of lenalidomide between April and June 2013, which was eventually stopped 2nd to renal toxicity and substantial improvement of the anemia. His anemia was ultimately attributed to pure red cell aplasia, which was caused by ipilimumab and resolved over a period of 6 months despite documentation of 5q- myelodysplastic syndrome.

**Fig. 2:**
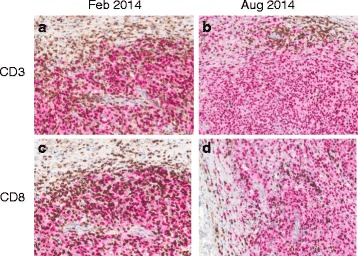
Melanoma surgical resection specimens were co-stained for CD3/SOX10 and CD8/SOX10, respectively. **a** CD3/SOX10, **c**, CD8/SOX10 staining of tumor resected in Feb of 2014. **b** CD3/SOX10, **d**, CD8/SOX10 staining of tumor resected in August of 2014

Between December 2012 and December 2013 there was continued slow growth of the right lower extremity metastases. By January 2014 accelerated progression of disease with substantial increase in the size of pre-existent right lower extremity skin nodules as well as development of new nodules was noted. A nodule in the lateral right popliteal area enlarged to a size of ca. 4 cm over a few weeks and became ulcerated and chronically infected. The patient was not a candidate for a clinical trial using PD-1/PD-L1 blockade. Given the lack of distant metastatic disease and the absence of compelling systemic treatment options, in February 2014 a palliative resection of the fast growing dominant nodule was performed. The patient had a complicated postoperative course with wound dehiscence and recurring infections requiring intense wound care over a period of 3 months. During the protracted postoperative course there was further growth of multiple skin metastases with emergence of new lesions both clinically and on restaging scans while no distant metastases were evident. Another palliative resection of a fast growing nodule on the right medial knee was performed in August 2014. By November 2014, almost 2 years after the first treatment with ipilimumab and 9 months after the first of 2 palliative resections, the patient noted shrinkage of several skin nodules. A restaging PET/CT performed in December 2014 showed a mixed response. By March 2015 (2 years after completion of ipilimumab) all non-resected in transit metastases had completely disappeared clinically; radiographically, in May 2015 there was a continued mixed response with most lesions reduced in size. By August 2016, all in transit metastases had resolved both clinically and radiographically and the patient remains in a complete clinical and radiographic response (Fig. 1c, d). Dual immunohistochemical staining of CD3 and CD8 with the melanoma marker SOX10 demonstrated brisk infiltration with CD3+ and CD8+ T cells in both surgically resected in transit metastases, suggesting a T cell mediated immune response against the tumor in both samples (Fig. 2). The metastasis which was resected in February 2014 was highly necrotic with some areas of viable tumor as evident by SOX10 staining, whereas no necrosis was seen in the metastasis that was removed in August 2014.

## Conclusion

We describe the case of an 84 year old man who presented with multiple in transit melanoma metastases on his right lower extremity that did not respond to ipilimumab, but eventually completely regressed, almost 2 years after the initial dose of ipilimumab, following two serial palliative resections of dominant masses that had became ulcerated and painful. Delayed tumor responses have been reported in melanoma patients treated with ipilimumab. The observation of delayed response kinetics and unusual response patterns with immunotherapy have lead to the development of immune related response criteria [5, 6]. These response patterns can include transient increases in baseline tumor lesions or development of new metastases. Nevertheless, a delay of almost 2 years between the first ipilimumab dose and the regression of in transit metastases would be highly unusual. The fact that the tumor responses were observed shortly after the palliative resection that resulted in infectious wound healing complications suggests that a tumor specific immune response was triggered by these interventions. While spontaneous regression of primary melanomas is not an uncommon event, spontaneous regression of metastatic melanoma is exceedingly rare [7, 8]. A report by Kalialis et al. collected only 76 cases from the literature since 1866 [8]. Operative trauma has previously been associated with spontaneous regression of metastatic melanoma lesions. Furthermore, it is known that regression of cancers can occur in the context of infection, especially those caused by Streptococcus pyogenes, first reported by William Coley. Mimicking this situation deliberately by injecting patients with streptococcus lead to regression of sarcomas in a case series [9]. These early observations have been interpreted as the initial evidence for the potential of immunotherapy as a treatment for cancer. Notably in a review of 68 melanoma regression cases, 21 were led by a febrile episode, of which 9 were associated to erysipelas [10].

Although the exact cause of our patient’s sudden onset of tumor regression, eventually leading to disappearance of all clinically and radiographically evident tumors, remains speculative, an immune related mechanism seems most plausible. We hypothesize that the operative trauma followed by the postoperative infections triggered an innate immune response similiar to a microbial immune adjuvant such as a Toll Like Receptor or a STING agonist [11, 12]. Notably, these and other strategies that are aimed at de novo induction of inflammation in the tumor such as radiotherapy or the recently approved oncolytic virus Talimogene Laherparepvec (T-VEC), which have provided evidence for systemic anti-tumor activity and are in clinical investigation either as monotherapy or with immune checkpoint inhibition [13, 14]. For our patient, it is intriguing to speculate that his exposure to ipilimumab almost 2 years prior to the onset of tumor regression also contributed to the tumor response.

## Methods

Dual immunohistochemical staining of CD3 (DAKO, A0452, 1:250) and CD8 (Dako, M7103, 1:200) with the melanoma marker SOX10 (EP 268, Cell Marque, 1:1500) was performed using an automated staining system (Bond III, Leica Biosystems, Buffalo Grove, IL) according to the manufacturer’s protocol, as previously described [15].

## Abbreviations

COPD: Chronic Obstructive Pulmonary Disease
CT: Computerized tomography
CTLA-4: Cytotoxic lymphocyte antigen-4
FDA: Food and Drug Administration
MRI: Magnetic Resonance Imaging
PD-1: Programmed death-1
PD-L1: Programmed death ligand 1
PET/CT: Positron emission tomography computerized tomography
T-VEC: Talimogene Laherparepvec
